# Potential Environmental Reservoirs of *Candida auris*: A Systematic Review

**DOI:** 10.3390/jof10050336

**Published:** 2024-05-08

**Authors:** Isabel Silva, Isabel M. Miranda, Sofia Costa-de-Oliveira

**Affiliations:** 1 Faculty of Medicine, University of Porto, 4200-319 Porto, Portugal; isabelfpsilva@gmail.com; 2 Cardiovascular R&D Centre UnIC@RISE, Department of Surgery and Physiology, Faculty of Medicine, University of Porto, 4200-319 Porto, Portugal; imiranda@med.up.pt; 3 Division of Microbiology, Department of Pathology, Faculty of Medicine, University of Porto, 4200-319 Porto, Portugal; 4 Center for Health Technology and Services Research—CINTESIS@RISE, Faculty of Medicine, University of Porto, 4200-319 Porto, Portugal

**Keywords:** *Candida auris*, environmental reservoirs, hospital environments, healthcare-associated infections

## Abstract

*Candida auris*, a multidrug-resistant yeast, poses significant challenges in healthcare settings worldwide. Understanding its environmental reservoirs is crucial for effective control strategies. This systematic review aimed to review the literature regarding the natural and environmental reservoirs of *C. auris*. Following the PRISMA guidelines, published studies until October 2023 were searched in three databases: PubMed, Web of Science, and Scopus. Information regarding the origin, sampling procedure, methods for laboratory identification, and antifungal susceptibility was collected and analyzed. Thirty-three studies published between 2016 and 2023 in 15 countries were included and analyzed. *C. auris* was detected in various environments, including wastewater treatment plants, hospital patient care surfaces, and natural environments such as salt marshes, sand, seawater, estuaries, apples, and dogs. Detection methods varied, with molecular techniques often used alongside culture. Susceptibility profiles revealed resistance patterns. Phylogenetic studies highlight the potential of environmental strains to influence clinical infections. Despite methodological heterogeneity, this review provides valuable information for future research and highlights the need for standardized sampling and detection protocols to mitigate *C. auris* transmission.

## 1. Introduction

**Figure 1:**
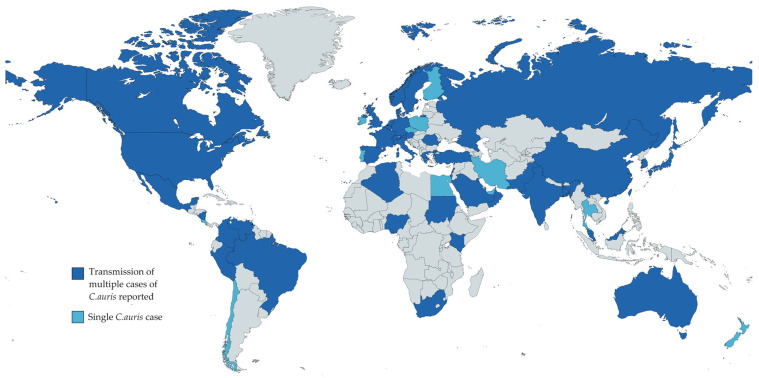
Global epidemiology of *Candida auris* until October 2023 [3,4,7,8,9,10,11,12,13,14,15,16,17,18]. The grey color represents countries with no *C. auris* cases published in the literature.

*Candida auris* is a multidrug-resistant yeast first identified in the ear of a Japanese patient in 2019 [1]. A member of the *Candida*/*Clavispora* clade, closely related to *Candida lusitaniae* and *Candida haemulonii*, it has emerged in recent years as a cause for healthcare-related infections, complicating treatment protocols and increasing patient morbidity and mortality rates [2]. It has been identified in more than 40 countries, with outbreaks reported on multiple continents, with notable impacts in countries such as the United States, India, South Africa, and several European nations, including the United Kingdom Spain, France, and Italy (Figure 1) [3,4]. In 2022, the World Health Organization designated this fungal pathogen as a priority due to its rapid transmission in healthcare settings and high mortality rate [5,6].

*C. auris* is implicated in a range of serious conditions including bloodstream and urinary tract infections, otitis, post-surgical complications, skin abscesses often related to catheter use, myocarditis, meningitis, osteomyelitis, and various wound infections [19]. However, it can also colonize noninvasive body sites, like skin, without causing active infection. Invasive infections caused by *C. auris* pose a significant mortality risk in hospital settings, with death rates varying between 30% and 60% [20]. Certain factors increase the likelihood of *C. auris* infection, including male gender, premature birth history in infants, underlying health conditions such as diabetes, kidney or hearing impairments, physical injuries, previous central venous catheter insertions, and extensive use of broad-spectrum antibiotics [21].

Phylogenetic studies obtained by whole-genome sequencing (WGS) techniques have identified four distinct clades, namely, clades I, II, III, and IV (South Asia, East Asia, Africa, and South America, respectively) [22]. A fifth and a sixth clade, separated from the others by more than 36,000 single-nucleotide polymorphisms (SNPs), were confirmed to have appeared in patients from Iran and Singapore, respectively [23,24].

*C. auris* has become a significant concern due to its multidrug-resistant nature. Of *C. auris* isolates, 44.29% are resistant to fluconazole, followed by 15.46% to amphotericin B, 12.67% to voriconazole, 3.48% to caspofungin, and 1.95% to flucytosine [25]. In the United States, the prevalence of resistance among these fungal pathogens has been observed to be 90% against fluconazole, 30% against amphotericin B, and 5% against echinocandins, with a tendency to rise over time [26,27]. Although resistance rates vary depending on the clade, country, and healthcare setting, *C. auris* has shown resistance to fluconazole, amphotericin B, and echinocandins [6,28].

Identifying *C. auris* isolates through conventional laboratory methods has proven to be challenging, often leading to its misidentification [4]. As a result, matrix-assisted laser desorption/ionization–time of flight (MALDI-TOF) and molecular identification via sequencing the ITS and/or D1/D2 DNA sequence seem to be good options for its identification [25].

*C. auris* thrives in environments with elevated salinity and high temperature, ideally between 37 °C and 40 °C [29,30]. Yeasts typically possess a keen sensitivity to ambient temperatures, often displaying a diminished ability to survive at the elevated temperatures in the human body [31]. The rise in global temperatures may have caused certain species, such as *C. auris*, to evolve and thrive in warmer climates [32]. This evolutionary development has allowed for *C. auris* to overcome the human body’s innate temperature defenses, thereby enabling colonization and leading to subsequent infection.

*C. auris* can exhibit the capacity to persist for extended periods on various surfaces due to its ability to form biofilms, like glass, fabric, plastic, wood, and steel, tending to flourish in moist environments [33]. Consequently, these materials pose a potential source of infection within hospital settings, emphasizing the importance of thorough disinfection protocols to interrupt the transmission of *C. auris* [4]. The significance of microbiological sampling of healthcare environment surfaces is emphasized in the literature, as it aids in understanding contamination levels and informs cleaning protocols. General recommendations have been proposed for designing routine protocols for surface sampling in healthcare environments [34].

The aim of this paper was to undertake a thorough systematic review to clarify the environmental reservoirs of *C. auris* in natural and hospital settings. Recognizing the environmental reservoirs of *C. auris* is a crucial aspect of comprehending pathogen transmission and will contribute to understanding the dynamics of the disease, leading to the development of more effective control strategies.

## 2. Materials and Methods

This systematic review was carried out in strict accordance with the *Cochrane Handbook for Systematic Reviews of Interventions* [35] and the Preferred Reporting Items for Systematic Reviews and Meta-Analyses (PRISMA) [36] checklist of the review was followed (Supplementary Material—S1).

### 2.1. Data Source and Search Strategy

The electronic bibliographic databases of PubMed, Scopus, and Web of Science were searched using a combination of MeSH terms and keywords: (“*Candida auris*” OR “*C. auris*”) AND (“environment” OR “environmental” OR “globalization” OR vector* OR “travel” OR “imported” OR “ecological” OR reservoir* OR “One Health” OR Ecosystem* OR “Climate Change” OR “Global warming” OR Niche*)

Studies were selected and screened until October 2023. The search included all publication types except reviews or systematic reviews, and no language restrictions were applied.

### 2.2. Eligibility Criteria

Studies were included in this review if they met the following criteria: (1) *C. auris* detected and isolated from natural environments or animals; (2) studies identified the presence of *C. auris* in urban environments outside of a hospital setting; (3) studies reporting the presence of *C. auris* from hospital environmental surfaces or materials. The exclusion criteria were (1) studies regarding hospital disinfection procedures or surface decontamination efficacy, (2) studies not addressing the detection of *C. auris*, and (3) systematic reviews, editorials, and grey literature.

### 2.3. Data Extraction and Synthesis

**Figure 2:**
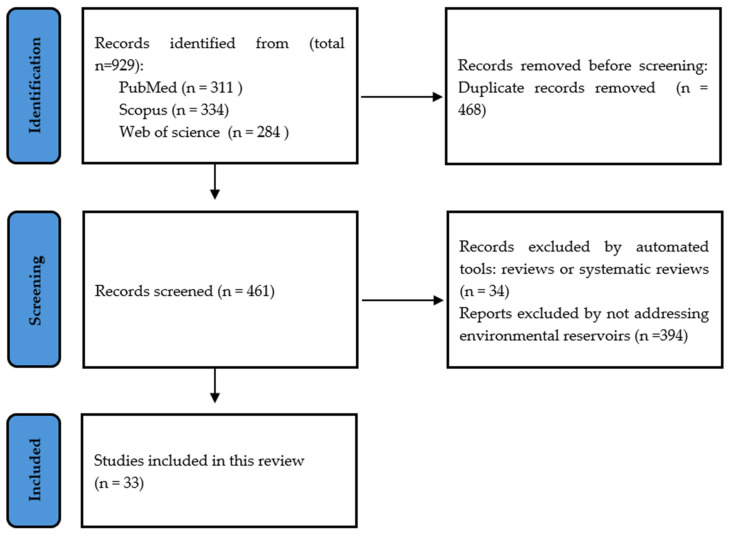
PRISMA flow chart representing the systematic identification of studies search via databases and registers.

A total of 929 references were obtained in the three databases. The extracted studies were uploaded to EndNote and Rayyan software [37] for duplicate removal, quality assessment, and further selection. In a blinded, standardized manner, a screening of the title, abstract, and full text was performed and guided based on inclusion and exclusion criteria by two independent reviewers (IS and SCO). Thirty-three studies were included in the systematic review (Figure 2).

A protocol was defined to synthesize the data collected from the selected studies uniformly and consistently. Data such as year of publication, country, study time frame, sample type, collecting procedure, laboratory methods for strain identification, clade determination, and susceptibility profile were extracted from the included publications. IS and SCO extracted the above information independently.

### 2.4. Risk of Bias (ROB) Assessment

To evaluate the risk of bias in the studies included in this review, the National Institute of Health (NIH) Quality Assessment Tool for Observational Cohort and Cross-Sectional Studies was used [38]. A 3-point scale was used to grade the potential source of bias as good, fair, or poor. No studies were excluded based on quality. ROB assessment was performed independently by SCO and IMM.

## 3. Results

Table 1 and Table 2 show the characteristics of the 33 studies included in this analysis, spanning the publication years from 2016 to 2023 and conducted within a sampling timeframe extending from 2013 to 2022. These studies were carried out across 15 countries, namely, the United Kingdom, the United States of America, Qatar, South Africa, Colombia, Spain, Kuwait, Oman, India, France, Hong Kong, Greece, China, the Netherlands, and the Republic of Korea.

Among the 33 studies included, 4 centered on natural environments, 3 examined urban non-hospital environments, 1 centered on both (Table 1), and 25 focused on isolation of *C. auris* within hospital settings (Table 2).

### 3.1. Potential Natural Environmental Reservoirs

Regarding the five studies that investigated *C. auris* in natural environments, four of these articles employed a similar approach for strain identification, beginning with its isolation through culture techniques and subsequent confirmation through genomic amplification [39,40,41,42]. The only study that deviated from this method attempted to identify previously misidentified *C. auris* strains from the Sequence Read Archive at the National Center for Biotechnology Information [43]. Generally, the collected samples were inoculated in Sabouraud Dextrose Agar, CHROMagar *Candida*, Yeast nitrogen, and Malt Extract agar at 24–42 °C.

*C. auris* was detected in diverse environmental substrates, including ears and skin from dogs [39], stored apples purchased from vendors [40], soil from a salt marsh, beach sand, and seawater in India [41], as well as water from estuaries in Colombia [42]. Genomic amplification alone enabled the identification of *C. auris* in unique contexts, such as on the skin of *Lissotriron vulgaris* and *Triturus cristatus* in a natural reserve in the UK, in the ear of a dog with otitis in Spain, and in airborne dust from Kuwait [43]. Among the reviewed studies, only the three conducted in India identified the clade of *C. auris* (South Asian clade) [39,40,41].

### 3.2. Potential Urban Non-Hospital Environmental Reservoirs

Out of the 33 articles included, only 4 reported the identification of *C. auris* in urban non-hospital environments [43,44,45,46]. Two of these studies solely relied on genomic amplification for identification, without performing culture [43,44]. The remaining two studies combined culture and PCR techniques [45,46]. They utilized CHROMagar medium at 42 °C, Sabouraud Dextrose Agar at 24 °C, and Malt Extract Agar at 24 °C for culture, successfully identifying *C. auris* in wastewater from various sources in Florida [45] and swimming pools in the Netherlands [46]. The other two studies identified *C. auris* solely through PCR, detecting its presence in activated sludge and membrane biofilm from a wastewater treatment plant in the Republic of Korea [43] and in pelleted solids from wastewater in the state of South Nevada, USA [44].

### 3.3. Potential Hospital Environmental Reservoirs

The other 25 papers focused on hospital environmental sources of *C. auris*, mostly during outbreaks of this fungal infection. As shown in Table 2, the studies employed various techniques for obtaining the samples. Sixteen studies used swabs [48,49,50,51,52,53,55,56,57,58,60,63,65,68,69,70], while two used gauze [54,59], and one used both direct contact plates and swab techniques [61]. Six studies did not report the methods of sample collection [13,47,62,64,66,67]. Among the studies that employed swabs, only 12 reported the material, of which 9 were sponges [48,55,58,60,63,65,68,69,70] and 3 were cotton [49,51,53]. All of the studies performed culture and subsequent confirmation through genomic amplification. The collected samples were inoculated in some Yeast Extract–Peptone–DextroseMedium, Yeast nitrogen enrichment, Sabouraud Dextrose agar or broth, Brilliance *Candida* agar, CHROMagar *Candida*, and Sabouraud ulcitol agar at 35–42 ºC. Only one study did not have culture-positive samples, having only identified *C. auris* by qPCR [49]. All of the studies included revealed that *C. auris* was frequently detected on surfaces near the patient’s bed area or in high-touch objects. Mobile medical devices, such as thermometers and stethoscopes, were contaminated with *C. auris* in 10 studies [48,50,53,54,55,59,60,61,65,69]. Additionally, 13 studies reported the presence of this yeast in areas outside the patient’s rooms, such as keyboards, sinks, and air vents [48,50,53,54,55,57,58,59,60,64,65,69,70]. The clade of *C. auris* was determined by WGS and analyzed by fifteen studies, of which twelve studies found the South Asian clade [13,48,49,50,51,52,55,56,57,62,63,66] and three studies detected the African clade [47,61,64].

Eleven studies tried to understand the relationship between clinical and environmental samples [13,47,48,50,51,57,60,61,63,64,66]. Five studies proved that they were similar [13,57,60,63,66], four that there was a close relationship between them [47,48,51,61], and two mentioned that there was a difference of between 1 and 160 SNPs between clinical and environmental samples [50,64].

### 3.4. Susceptibility of Natural and Hospital Environmental Samples

Table 3 presents the susceptibility profiles to various antifungal agents commonly used and gene mutations responsible for reduced susceptibility in all studies included in this analysis. Among the studies included, eleven tested the susceptibility profiles for antifungals, with four conducted in natural environments [39,40,41,42] and seven in hospital settings [47,50,51,56,57,60,69]. Of these, nine studies utilized the Clinical & Laboratory Standards Institute broth microdilution method to estimate minimum inhibitory concentrations [39,40,41,42,50,51,56,69], one employed the MICRONAUT-AM broth microdilution procedure [57], one used Sensititre YeastOne plates [47], and one did not specify the method used [60]. Although there are currently no established susceptibility breakpoints specific for *C. auris*, and there is still no clear correlation between microbiologic breakpoints and clinical outcomes, the CDC has proposed tentative minimal inhibitory concentration (MIC) breakpoints for fluconazole (≥32 mg/L), amphotericin B (≥2.0 mg/L), and anidulafungin (≥4 mg/L) based on closely related *Candida* species and expert opinion [71].

The susceptibility profile of the strains isolated from natural environmental samples to antifungal drugs was tested in four studies. Arora and colleagues found that sand/beach samples had high MIC values for fluconazole (MIC > 32 mg/L) and amphotericin B (>2 mg/L), but soil samples from the salt marsh had low MIC values for both drugs [41]. Salt marsh samples also grew slower and preferred lower temperatures than the others. Apple samples stored in a warehouse showed low MIC values for amphotericin B (<2 mg/L), but fluconazole MIC values varied [40]. Water samples from Colombian estuaries had low MIC values for fluconazole and amphotericin B [42]. Samples from dog’s ears and skin showed high MIC values for fluconazole and low for amphotericin [39]. All samples showed low MICs to anidulafungin across all studies.

Three studies investigated genetic mutations linked to antifungal resistance [39,40,41]. These studies identified mutations in the *CDR1*, *ERG11*, and *TAC1B* genes. Specifically, the *CDR1* gene revealed V704L and E709D mutations [40], the *TAC1B* gene showed an A640V mutation [39], and the *ERG11* gene exhibited K143R [39,40] and Y132F mutations [40,41].

Seven studies tested the susceptibility of hospital environmental samples and found that all samples tested showed low MIC values to anidulafungin [47,50,51,56,57,60,63,69]. Except for Al Maani et al.’s study [56], all the other articles had samples that showed high MIC values for fluconazole (MIC ≥ 32 mg/L). Out of these, only one contained samples with elevated MIC values exclusively [47]. Only one study included samples that exclusively exhibited low MIC values for amphotericin B [47]. Five studies reported samples with high MIC values of amphotericin B (MIC ≥ 2.0 mg/L) [50,51,56,60,69]. In one study, MIC values for fluconazole and amphotericin B were not provided; instead, they were classified as resistant and susceptible, respectively [57]. Five studies examined genetic mutations associated with resistance to certain antifungals [47,50,51,57,63]. Mutations in the *ERG11* gene were identified, particularly VF125AL (I74L) [47], Y132F [50,51,57], and K143R [63].

### 3.5. Risk of Bias—Quality Assessment

Due to the heterogenicity of the studies regarding natural environments, quality assessment was performed only in hospital environmental studies. All analyzed studies were classified as “Good”, being methodologically robust, with clear objectives, thorough methods for sample collection and analysis, and significant contributions to understanding the molecular epidemiology and drug resistance (Supplementary Material—S2).

## 4. Discussion

The present systematic review, conducted per PRISMA guidelines, provides an update on potential environmental reservoirs of *C. auris*. This is necessary to understand the disease’s epidemiology and important niches, which in turn helps with its prevention and management.

### 4.1. Sampling and Detection

Our study’s findings highlight the differences concerning environmental sampling procedures found both within and across nations. Few studies have compared the most efficient method for environmental sampling recovery for *Candida auris*. One study found that culture recovery is best with swabs instead of sponges, with 8.4% and >0.1% in culture recovery, respectively [72]. Additionally, using a polyurethane sponge seems to be better for *C. auris* recovery than cellulose sponges. Another article has proven that inoculating *C. auris* samples in Salt Sabouraud Enrichment Broth is better for *C. auris* isolation than directly plating it in CHROMagar [30]. The enrichment broth procedure inhibits the growth of another organism by creating a high-temperature and high-salinity environment, so it should only be used when trying to isolate *C. auris* specifically. Most studies in this review used both culture and molecular/proteomic techniques to detect and identify *C. auris* in the environment. One of the most effective identification methods used for detecting *C. auris* in cultured isolates is MALDI-TOF MS, which reduces the processing period to less than three hours compared to traditional and DNA-based methods [73]. Researchers can use the Bruker and Vitek MALDI-TOF MS systems to correctly identify *C. auris* [74]. Vitek 2 technology, with software version 8.01, may misidentify *C. auris* as *C. duobushaemulonii* [74]. Therefore, additional testing is necessary to rule out *C. auris* when using this technology. A reliable way to distinguish *C. auris* from other yeast species is to sequence the rDNA regions (internal transcribed spacer and D1/D2 region of large subunit) [75]. PCR/real-time PCR assays are a dependable method to rule out negative samples when additional analysis is required for further investigation [76].

### 4.2. Potential Natural Environmental Reservoirs

As shown in Table 1, *C. auris* was isolated from a wide range of natural habitats, indicating a potential reservoir of *C. auris* in the environment and/or intermediate host. According to reports from two countries located on separate continents at the time of this analysis, aquatic environments appear to be a source of *C. auris*. Furthermore, amphibians known to live in aquatic environments have been shown to contain this yeast’s DNA. These findings imply that numerous hosts can become contaminated by the aquatic environment and may play a role in *C. auris*’ persistence in this habitat, like other yeasts.

This analysis reports the occurrence of *C. auris* infections in animals, which is consistent with the literature documenting similar cases caused by other *Candida* species [77]. In humans, the majority of *Candida* infections arise from colonizing strains rather than through transmission, rendering the likelihood of zoonotic disease minimal [78]. Even with the diminished likelihood of zoonotic transmission, animals could still play a role in disseminating *C. auris* within the environment, which may subsequently result in human colonization.

Researchers have found *C. auris* on the surfaces of apples bought in local food markets in India, but not on freshly harvested apples from orchards [79]. This indicates that contamination with *C. auris* may occur during transportation or storage. Identifying the stage at which contamination occurs may be important in understanding its impact and risk to human contamination and subsequent infection.

Finding *C. auris* in aquatic environments supports the hypothesis that it may have natural reservoirs outside human-made environments. The presence of *C. auris* in these settings suggests that it might have adapted to survive or even thrive in aquatic conditions. Notably, isolation of the drug-susceptible *C. auris* strain from aquatic habitat with no known human activity probably indicates that *C. auris* existed as a drug-susceptible pathogen and developed multidrug-resistant traits after its adaptation in humans [41].

### 4.3. Potential Urban Non-Hospital Environmental Reservoirs

*C. auris*, known to colonize the skin and found in human excrement, has the potential to infiltrate plumbing systems via skin shedding and fecal disposal, eventually arriving at wastewater treatment facilities [80]. It is, therefore, unsurprising that it has been identified in such facilities and sewage systems across multiple countries. This situation, however, presents certain challenges. Firstly, sewage discharge can facilitate the propagation of microbes, including *C. auris,* into marine ecosystems due to the coastal and estuarine outflows of wastewater treatment plants and sewer overflow [81,82]. Secondly, in the face of water shortages, the prospect of utilizing wastewater effluents for irrigation and drinking water production is being explored [83]. This could potentially amplify the spread of microbiological agent into the environment, thus requiring careful management strategies [84].

One study reported finding *C. auris* in air dust samples from Kuwait. Previous research showed that airborne microorganisms can travel far and settle on human skin, affecting human health and causing diseases [85,86]. *C. auris* was also found in swimming pools in Netherlands; it is known that recreational swimming pools can also pose the threat of water-borne yeast infection, particularly in immunocompromised patients [87].

### 4.4. Potential Hospital Environmental Reservoirs

The role of hospital surfaces in the transmission of healthcare-associated infection has been highlighted by several authors [88]. Environmental surfaces that are close to or frequently touched by colonized patients are more likely to harbor these organisms [89]. Nevertheless, we should be aware that most of the studies included were conducted in the context of outbreak investigations, and the level of contamination in non-outbreak situations remains uncertain. While it is true that five studies found that the environmental samples were identical to the clinical samples, it is important to note that they did not establish a correlation between this contamination of the environment and the transmission of *C. auris* to other patients. Only one study explicitly correlated the contamination of the environment (temperature probes) as a source of transmission of *C. auris*, leading to an outbreak [61]. Therefore, it is essential to apply strict hygiene measures and disinfect the environment using an Environmental Protection Agency-registered hospital-grade disinfectant proven effective against *C. auris* or disinfectants known to be effective against *Clostridium difficile* spores to avoid transmission [90,91,92].

This yeast was also detected in areas not within reach by humans, such as air vents and very high curtains. Like *Acinetobacter* and *Staphylococcus aureus*, *C. auris* may disperse through the air by shedding skin flakes that contain the fungus and are carried by air currents [93,94]. It was also found outside patients’ rooms, and on computer keyboards and medical carts, which may suggest that the medical staff are not sanitizing their hands properly [89,90].

### 4.5. Susceptibility and Phylogeny

*C. auris* poses a significant challenge within the domain of mycotic diseases due to its resistance to multiple drugs and complicating therapeutic approaches [20]. Echinocandin treatment is considered the first-line option [95]. However, in cases of echinocandin resistance, treating *C. auris* infections often involves combination antifungal therapy. Combining echinocandins with other antifungal agents such as triazoles or polyenes may enhance efficacy, broaden the antifungal spectrum, and reduce the potential for resistance development [20]. To ensure effective control of *C. auris* infections, diligent patient monitoring must be observed for signs of clinical recovery during antifungal therapy.

Of the included studies in this review, gene sequencing for various isolates showed mutations in *ERG11* and *TAC1B*, known to contribute to reduced azole susceptibility [96,97], and showed mutations in the *CDR1* gene: these changes are known to contribute to antifungal resistance [98]. In addition to the mutations identified in the included studies, other relevant ones are highlighted in the literature. Mutations in the *ERG3* and *ERG5* genes have been found to impact susceptibility to amphotericin B, while mutations in the *FKS1* gene affect susceptibility to echinocandins [20].

Clades I, III, and IV are associated with outbreaks of invasive and multidrug-resistant infections [99]. Clade II frequently exhibits susceptibility to all antifungal medications, with the majority of instances presenting as ear infections. To date, there have been no associations with outbreaks. Clade II strains exhibit a different karyotype, with large sub-telomeric deletions and rearrangements, and are less commonly linked to invasive infections. Clade V and VI isolates have not been extensively studied, and their clinical implications remain to be fully understood [23,24].

### 4.6. Strategic Surveillance Approach

Based on our findings, it is evident that *C. auris* frequently contaminate areas surrounding the patient’s bed. Therefore, to assess effective disinfection, priority should be given to these zones, with particular attention to bedside tables and hand-rails. Additionally, mobile medical devices should be included in routine investigations due to their potential for cross-contamination between patients [61]. Ensuring proper disinfection protocols for these devices with proper hand hygiene measures is essential in minimizing the risk of transmission of pathogens, including *C. auris* [100].

In terms of environmental sampling for *C. auris*, an effective method would involve swabbing surfaces [72] and inoculating them in Salt Sabouraud Enrichment Broth [30], followed by MALDI-TOF MS or PCR analysis [76]. Positive samples should then undergo culture to confirm viability and perform antifungal susceptibility testing. This comprehensive approach allows for the detection of viable fungal organisms and ensures an accurate assessment of environmental contamination levels.

### 4.7. Limitations

This study has some important limitations that should be noted. A limited number of studies has been conducted on isolation in natural settings, and therefore, this review can only offer a snapshot of the potential reality. Choosing where to collect samples in an environment can significantly impact the relevance and applicability of the data obtained. Another limitation is the heterogeneity of the methods used in the articles that were reviewed, such as the study design and the environmental sampling techniques for *C. auris* detection. Also, these differences could affect the consistency and validity of the results and the implications derived from them. Furthermore, the research had some limitations that prevented a comprehensive understanding of the reservoir behavior, such as the inability to identify the strain involved and not performing susceptibility assays. It is important to emphasize that only studies regarding the hospital environmental detection of *C. auris* in the context of an outbreak were considered.

## 5. Conclusions

Despite these limitations, the present review demonstrates potential environmental reservoirs of *C. auris* in clinical and non-clinical environments. In addition, it offers a broad perspective on various aspects that need more research, providing guidance for future studies on *C. auris*.

It is worth mentioning that global warming may pose a substantial health risks concerning the proliferation of fungal infections [101]. The gradual increase in temperature promotes an environment that favors the evolution of yeasts, allowing for them to acquire thermotolerance [102]. This adaptation allows for a broader geographic distribution of pathogenic yeasts and their carriers since warmer climates make previously inhospitable regions susceptible to fungal diseases [101]. Climate-induced phenomena such as floods, storms, and hurricanes increase the risk of fungal infections. These events have the potential to spread fungal spores on a large scale and aerosolize them, which increases the possibility of human contact. In addition, the injuries caused by these disasters serve as a gateway for fungal pathogens, introducing new or uncommon species into humans [101]. Therefore, the continued rise in global temperatures may lead to an expansion of the natural habitat of *C. auris*, further increasing the risk of this infection in humans [103]. Tackling the interplay between climate change and fungal infections, including *C. auris*, requires proactive measures to mitigate climate change and improve surveillance, prevention, and control strategies for emerging fungal pathogens.

## Figures and Tables

**Table 1:** Potential non-hospital environmental reservoirs of *C. auris*.

First Author and Year	Country	Time of the Year	Positive Type of Samples	Harvesting Method	Culture and Temperature	Genomic Amplification	Proteomic	Clade
Yadav, 2022 [40]	India	March–April 2020; June–July 2021	Stored apples (epicarp)	Sterile Swabs Swept	Sabouraud dextrose with chloramphenicol and gentamicin, CHROMagar *Candida*, and Yeast nitrogen broth at 37 °C	Illumina Hiseq 4000	MALDI-TOF MS	I
				Epicard homogenizes in saline				
Yadav, 2023 [39]	India	-	Ear and skin of dogs	Swab samples	Yeast nitrogen broth with 10% NaCl at 37 °C for 4 days	Illumina HiSeq 4000	MALDI-TOF MS	I
Arora, 2021 [41]	India	February–March 2020	Salt march soil and sandy beach sediment	2 g sediment suspended in 8 mL of 0.85% NaCl	Sabouraud dextrose agar plates with chloramphenicol and gentamicin at 28 °C up to 7 days	Illumina Hiseq 4000	MALDI-TOF MS	I
			Seawater from the sandy beach	filtered with 0.45 um filters in 50 mL sterile bottles				
			Membrane biofilm					
Escandón, 2022 [42]	Colombia	2018	Water from Estuaries	100 mL of water from a depth of 30 cm	Salt Sabouraud Dextrose selective broth at 40 °C for 48 h	PCR	MALDI-TOF MS	-
Irinyi, 2022 [43]	UK	2019	Skin of *Lissotriron vulgaris* and *Triturus cristatus*	Sterile Swabs	-	Illumina Miseq	-	-
	Spain	2019	Ear of dog	-	-	PCR	-	-
	Kuwait	2017–2018	Airborne dust	Dust-laden filter paper from a high-volume air sampler	-	Illumina HiSeq 2500	-	-
	Republic of Korea	2016	Activated sludge	50 mL of a sterilized conical tube	-	Illumina MiSeq	-	-
Barber, 2023 [44]	USA	June–September 2022	Pelleted wastewater solids	-	-	qPCR	-	-
Babler, 2023 [45]	USA	2021–2022	Wastewater from the central district plant	Sterile HDPE bottle containing 0.1 g sodium thiosulfate	CHROMagar at 42 °C for 48 h	qPCR	-	-
			Wastewater from sewer cleanout from the hospital					
Ekowati, 2018 [46]	Netherlands	-	Water samples from two pools	Plastic containers	Sabouraud Dextrose Agar and Malt Extract Agar at 24 °C for 7 days	PCR	-	-

Polymerase chain reaction (PCR), quantitative polymerase chain reaction (qPCR).

**Table 2:** Sites of detection of *C. auris* in hospital environment.

First Author and Year	Country	Time of the Year	Sample Type	Sampling Method	Culture and Detection	Positive Samples/Total Samples Collected	Molecular Identification	Clade and Clinical Identical	Conclusion
Katsiari, 2023 [13]	Greece	October 2020–January 2022	Environmental screening process	-	Sabouraud Dextrose Agar at 35 °C and 42 °C	3/NA	PCR MALDI-TOF	Clade I and identical sequences to clinical samples	Only beds and a side table near infected patients were positive.
Tian, 2021 [47]	China	March 2018	Environmental screening process	-	Yeast Extract–Peptone–Dextrose Medium at 37 °C for 16 h	1/1	Illumina NovaSeq platform MALDI-TOF	Clade III clade and clinically related	Only the bedrails of a patient infected were positive.
Didik, 2023 [48]	Hong Kong	September–October 2022	Frequently touched items of ward communal area returned air grilles and high-level supply air grilles.	Flexible pre-moistened sterile poly wipe sponge swabs	Sabouraud dextrose broth with 10% NaCl, chloramphenicol and colistin for 7 days at 40°	32/249	Illumina iSeq or MiSeq MALDI-TOF	Clade I	29 positive samples were from frequently touched items and 3 from returned air grilles and supply air grilles.
Alanio, 2022 [49]	France	January 2021	Investigation of the environment after bio-cleaning (sodium hypochlorite and sporicide)	Sterile pre-moistened cotton swabs unloaded in water.	Sabouraud Dextrose Agar	0/NA	qPCR	Clade I	Only the mattresses, bed fences, and trolleys of infected patients were positive.
					qPCR	NA/NA			
Yadav, 2021 [50]	India	December 2019–May 2020	Near each patient’s bed (bed railing, bed sheet, pillow, bedside trolly, floor, and air conditioner air wings), medical equipment (thermometer, B.P. cuffs, ECG clip and sucker, oxygen mask, and nebulizer), and portable devices (mobile, wheelchair, and intravenous pole).	Premoistened swabs	Sabouraud Dextrose Agar containing chloramphenicol and gentamicin at 37 °C for 48–72 h	15/148	Illumina Hiseq 4000 MALDI-TOF	Clade I and SNP difference Between 1–160 among clinical and environmental isolates.	Recovered from near-patient sites (floor, bed railing, bedside trollies, pillow, and bed sheet). It was also recovered from air conditioner air wings, a mobile phone, and two medical equipment: an oxygen mask and intravenous pole.
					Yeast nitrogen enrichment broth containing 10% NaCl and 2% mannitol as a carbon source, and vortexed and incubated at 37 °C for 72–96 h				
Umamheshwari, 2021 [51]	India	December 2018–March 2019	Beds, bed rails, bedside tables/cardiac tables, and nursing cart.	Sterile pre-moistened in saline cotton swabs	Sabouraud Dextrose Agar plates for 7 days at 37 °C	2/46	PCR VITEK 2 MALDI TOF MS	Clade I and >90% similarity between clinical and environmental isolates	Only the bed railings around an infected patient were positive
Taori, 2019 [52]	UK	July 2016–February 2017	High touch point areas	Sterile pre-moistened in saline cotton swabs	Brilliance *Candida* agar for 48 h at 37 °C	2/48	PCR VITEK 2 MALDI TOF MS	Clade I	Only the bed railings and dining trolleys around an infected patient were positive
Biswal, 2017 [53]	India	January–March 2017	Environmental sampling of surfaces of objects or fomites in ICU	Cotton swabs pre-moistened in saline	Sabouraud dextrose agar for 48 h at 37 °C	24/304	Sequencing of ITS and D1/D2 regions of ribosomal DNA MALDI TOF	-	Recovered from near-patient sites (beds) and medical equipment: expiratory end of the ventilator, ECG leads, cuffs blood pressure, and temperature probes.
Ruiz-Gaitan, 2019 [54]	Spain	2016–2018	Patients’ environment (bed rails, table, infusion pumps, keyboards, and walls) after cleaning, faucets, benches, and reusable medical devices.	Cotton gauzes soaked in saline	Sabouraud dextrose broth with chloramphenicol at 35 °C for 72 h	61/738	Sequencing ITS VITEK MS IVD	-	Isolated from Blood pressure cuffs, patient tables, keyboards, infusion pumps
Adams, 2018 [55]	USA (New York)	2013–2017	Environmental samples were collected from rooms belonging to infected patients on near-patient surfaces, equipment, and other objects. They were also collected from objects outside these rooms.	Sponge sticks with 45 mL of phosphate-buffered saline with 0.02% Tween 80	Sponge suspension on different agar media or Sponge suspension in 5 mL of SDB-AS broth at 40 °C for 2 weeks	62/781	Real-time PCR MALDI-TOF	Clade I	Isolated from near-patient surfaces and other surfaces inside patients’ rooms, like floors, curtains, and others. It was also isolated from equipment outside these rooms, like vital sign machines, thermometers, and others.
					Real-time PCR	19/781 (culture negative)			
Al Maani, 2019 [56]	Oman	October 2018	Collected from high touch areas and re-useable devices	Sterile swabs pre-moistened in sterile saline	Sabouraud dextrose agar and incubated at 37 °C for 48 h.	2/140	PCR MALDI-TOF MS	Clade I	Isolated from the ventilator end and trolley belonging to the patient’s rooms.
Alfouzan, 2020 [57]	Kuwait	January 2018–2019	Collected from infected patients’ rooms.	Swab samples	Sabouraud dextrose agar with gentamicin for 24–48 h at 37 °C	7/261	PCR VITEK 2 MALDI TOF MS	Clade I and genetically identical to clinical strains	Isolated from the Bedrail, bedside drawer, toilet flush handle, toilet faucet handle, and wall
Kumar, 2019 [58]	USA	April–June 2017	Patients’ room objects <3 feet from the patient, >3 feet away, and bathrooms. As well as portable medical equipment.	Sponge sticks pre-moistened with neutralizing buffer	Sabouraud dextrose agar and incubated at 37 °C for 96 h.	8/204	MALDI-TOF	-	Isolated from the Bedrail, bedside table, call button, and sink drain
Ruiz-Gaitán, 2018 [59]	Spain	April 2016–January 2017	Environmental surveillance on various surfaces and objects.	Cotton gauze moistened with saline	Sabouraud dextrose agar with chloramphenicol For 72 h at 35 °C	-	PCR Vitek MS Ruo	-	Isolated from Beds, tables, floors, walls, keyboards blood pressure cuffs, and hemodialysis drains
Escandón, 2019 [60]	Colombia	February 2015–August 2016	Samples were collected from surfaces and objects belonging to four zones in patients’ rooms: zone 1 being near bed, zone 2 being infrequent patient contact, zone 3 with almost no contact, and zone 4 being bathrooms adjects to patients’ rooms.	3M Sponge sticks and EnviroMax Plus swabs	Salt Sabouraud dextrose broth	37/322	Illumina Hiseq 2500 MALDI-TOF	Genetically identical to clinical strains	From zone 1 it was isolated from bedrails, cellular phones, hand controllers, and floors. From zone 2, chairs, bed trays, and medical equipment. From zone 3, closets, door handles, and alcohol gel dispensers, and zone 4 sink basins, bedpans, and mop buckets.
Eyre, 2018 [61]	UK	November 2016–April 2017	Environmental screening	Bacterial swabs in a liquid transport medium (Sigma Transwab) and sponges to sample larger surface areas (Polywipe) and Sabouraud dextrose agar contact plates	Sabouraud Dextrose Agar with chloramphenicol at 37 °C	NA/128	Illumina miseq MALDI-TOF	Clade III and clinical and environmental samples were closely related.	Isolated from a pulse oximeter, temperature probes, and patient mobile hoist.
Rhodes, 2018 [62]	UK	April 2015–November 2016	Environmental screening of a room of a colonized patient.	-	Sabouraud Dextrose Agar at 35 °C for 18–48 h	2/2	Illumina hiseq 2500 MALDI-TOF	Clade I	Isolated from Beds and trolleys
Lesho, 2018 [63]	USA	-	3/132 (outside)	Sponge sticks and premoistened rayon-tipped swabs	Sabouraud Dextrose Agar with gentamycin and chloramphenicol 5 days	3/ 132	WGS MALDI-TOF	Clade I and genetically identical to clinical strains	Isolated from reclining chairs inside the patient room and sink outside the room.
Naicker, 2021 [64]	South Africa	2017	Hight touch surfaces and objects and other surfaces in patient care are	Swab	Sabourad agar	10/NA	WGS MALDI-TOF	Clade III and there were a maximum of 27 SNP differences between environmental to clinical strains.	Isolated from handwashing basin, bed linen, bed rails, window-sill, a curtain, drying rack, and the floor.
Pacilli, 2020 [65]	USA	May 2016–December 2018	Hight touch surfaces in patient care environments, multiuse patient care items, and mobile equipment.	3M Sponge sticks with neutralizing buffer, homogenized in 40 mL of phosphate-buffered saline with 0.02% Tween 80	CHROMagar *Candida* plates for 7 days	73/191	MALDI-TOF	-	Isolated from glucometers, temperature probes, mobile ultrasounds, pulse-oximeters, blood pressure cuffs, stethoscopes, over-bed tables, bedside chairs, nursing carts, doorknobs, bedrails, and windowsills
Salah, 2021 [66]	Qatar	April 2018–November 2020	Environmental screening	-	CHROMagar *Candida* for 5 days at 42 °C	-	Illumina NextSeq 550 or Illumina Miseq MALDI-TOF	Clade I and genetically identical to clinical isolates	Isolated from a bedside table, bed, couch, and cabinet inside patients’ rooms.
Schelenz, 2016 [67]	UK	April 2015–July 2016	Environmental screening of the area surrounding colonized patients	-	Sabouraud Dextrose Agar plates	-	MALDI-TOF	-	Isolated from the Floor around bedsites, trollies, radiators, windowsills, equipment monitors, keypads
Sexton, 2021 [68]	USA (Chicago)	October 2018	Environmental screening of patients’ rooms windowsills, doorknobs, and handrails.	3M Sponge sticks with neutralizing buffer, homogenized in 40 mL of phosphate-buffered saline with 0.02% Tween 80	CHROMagar *Candida* plates at 40 °C for 72 h	50/100	qPCR MALDI-TOF	-	Isolated from window, indoor knob, outdoor knob, left handrail and right handrail
					qPCR	70/100			
Zhu, 2020 [69]	USA	2016–2018	Environmental sampling of porous and nonporous surfaces.	3M sponge sticks, vortexed with 1 mL modified liquid amies medium	Sabouraud Dextrose Agar with chloramphenicol, gentamicin, penicillin and streptomycin, and Sabouraud ulcitol agar containing the above antibacterial with 10 salt	109/3672	PCR MALDI-TOF	Clade I	Isolated from near-patient environments (floors, beds, walls, etc.), from mobile medical equipment (lifter, blood pressure cuff, etc.), and outside rooms (computer keyboard). Degrees of colonization were significantly higher on nonporous than porous surfaces.
					PCR	434/3672			
Patterson, 2021 [70]	UK	-	-	3M Sponge sticks with neutralizing buffer	-	-	-	-	A patient’s bed space and staff lanyards were infected.

Whole-genome sequencing (WGS), polymerase chain reaction (PCR), quantitative polymerase chain reaction (qPCR).

**Table 3:** Results of antifungal susceptibility testing and gene mutations.

	First Author, Year	Gene Mutation			MIC (mg/L)/Phenotype		
		*CDR1*	*TAC1B*	*ERG11*	FCZ	AmB	AND
Natural Environment	Yadav, 2023 [39]	-	A640V (2/6)	K143R (2/6)	32–128	0.25–0.5	0.01–0.06
	Yadav, 2022 [40]	V704L (13/19) E709D (3/19)	-	K143R (13/19) Y132F (3/19)	16–128	0.25	-
	Escadón, 2022 [42]	-	-	-	2.0	0.5	0.25
	Arora, 2021 [41]	-	-	Y132F	8–256	1–4	0.125
Hospital Environment	Tian, 2021 [47]	-	-	VF125AL (I74L)	256	1	0.5
	Yadav, 2021 [50]	-	-	Y132F	16–128	0.25–4	0.125–0.5
	Umamaheshwari, 2021 [51]	-	-	Y132F	16–32	2	0.25–0.5
	Alfouzan, 2020 [57]	-	-	Y132F	R *	S **	-
	Al Maani, 2019 [56]	-	-	-	8–16	1–2	0.031
	Escadón, 2019 [60]	-	-	-	2–64	0.38–4	0.03–0.125
	Zhu, 2019 [69]	-	-	-	8–256	0.25–3.0	0.08–1
	Lesho, 2018 [63]	-	-	K143R (1/1)	-	-	

Minimum concentration inhibition (MIC); fluconazole (FCZ); anidulafungin (AND); amphotericin B (AmB); * resistance (R) to FCZ was set at MIC of ≥32 mg/L, AmB at ≥2.0 mg/L, AND at ≥4 mg/L [71]. ** Susceptible (S).

## Supplementary Materials

The following supporting information can be downloaded at: https://www.mdpi.com/article/10.3390/jof10050336/s1. Table S1. PRISMA checklist of the systematic search of the relevant studies. Table S2. Risk of Bias assessment of the included studies by NIH Quality Assessment Tool for Observational Cohort and Cross-Sectional Studies.
